# Effects of azithromycin on bronchial remodeling in the natural model of severe neutrophilic asthma in horses

**DOI:** 10.1038/s41598-021-03955-9

**Published:** 2022-01-10

**Authors:** Sophie Mainguy-Seers, Roxane Boivin, Sheila Pourali Dogaheh, Francis Beaudry, Pierre Hélie, Alvaro G. Bonilla, James G. Martin, Jean-Pierre Lavoie

**Affiliations:** 1 Department of Clinical Sciences, Faculty of Veterinary Medicine, Université de Montréal, St-Hyacinthe, QC J2S 2M2 Canada; 2 Department of Veterinary Biomedical Sciences, Faculty of Veterinary Medicine, Université de Montréal, St-Hyacinthe, QC J2S 2M2 Canada; 3 Department of Pathology and Microbiology, Faculty of Veterinary Medicine, Université de Montréal, St-Hyacinthe, QC J2S 2M2 Canada; 4 Meakins Christie Laboratories, McGill University, McGill University Health Center Research Institute, Montreal, QC H4A 3J1 Canada; 5 Present Address: Laboratoire de Sciences Judiciaires Et de Médecine Légale, Ministère de La Sécurité Publique, Montreal, QC H2K 3S7 Canada

**Keywords:** Asthma, Antimicrobial therapy, Combination drug therapy, Chronic inflammation, Respiration, Neutrophils, Chemokines

## Abstract

Steroid resistance in asthma has been associated with neutrophilic inflammation and severe manifestations of the disease. Macrolide add-on therapy can improve the quality of life and the exacerbation rate in refractory cases, possibly with greater effectiveness in neutrophilic phenotypes. The mechanisms leading to these beneficial effects are incompletely understood and whether macrolides potentiate the modulation of bronchial remodeling induced by inhaled corticosteroids (ICS) is unknown. The objective of this study was to determine if adding azithromycin to ICS leads to further improvement of lung function, airway inflammation and bronchial remodeling in severe asthma. The combination of azithromycin (10 mg/kg q48h PO) and inhaled fluticasone (2500 µg q12h) was compared to the sole administration of fluticasone for five months in a randomized blind trial where the lung function, airway inflammation and bronchial remodeling (histomorphometry of central and peripheral airways and endobronchial ultrasound) of horses with severe neutrophilic asthma were assessed. Although the proportional reduction of airway neutrophilia was significantly larger in the group receiving azithromycin, the lung function and the peripheral and central airway smooth muscle mass decreased similarly in both groups. Despite a better control of airway neutrophilia, azithromycin did not potentiate the other clinical effects of fluticasone.

## Introduction

Although asthma is uniformly described as a chronic inflammatory disease resulting in expiratory airflow limitation, it is a highly heterogeneous condition with a large spectrum of severity^1^. Despite affecting only ≈ 5–20% of asthmatic subjects^2,3^, severe asthma is a major burden considering its negative impact on quality of life and its considerable healthcare costs^4,5^. High-dose inhaled, or even oral, corticosteroids are often insufficient in these patients^3^, prompting the need for additional treatment options.

Airway neutrophilia has been associated to negative clinical outcomes including severe manifestations of the disease^6^, acute exacerbations^7^, persistent fixed airway obstruction^8^ and corticosteroid resistance^9^. The release of inflammatory mediators with the propensity to damage airway architecture, such as elastase^7^ and neutrophil extracellular traps^10^, partly explains why neutrophils are deemed harmful. However, whether these leukocytes are causative or simply bystanders in the natural history of asthma is speculative. Therefore, studying clinical outcomes when airway neutrophilia is controlled could help elucidate its involvement in asthma pathophysiology.

The macrolide antimicrobials have proven useful in respiratory conditions characterized by neutrophilic inflammation such as diffuse panbronchiolitis and chronic obstructive pulmonary disease^11^, and have been the focus of recent large clinical trials in asthma^12,13^. Notably, the prolonged use of azithromycin, combined with standard therapy, improved the quality of life and reduced the frequency of exacerbations in patients with uncontrolled asthma^12^. However, the mechanisms underlying these positive outcomes are unclear as the spectrum of action of macrolides goes beyond antimicrobial and immunomodulatory effects^11^. The modulation of airway smooth muscle (ASM) biology in vitro^14–17^ and the decrease of ASM mass in experimental models of asthma by macrolides^18–21^ might explain their attenuation of bronchial hyperresponsiveness^22,23^. However, these results need confirmation in humans as treatment success obtained in rodent models frequently fails to translate to clinical therapy^24^. Results of studies from species naturally affected by asthma may possibly better translate to humans^25^.

Asthma is a prevalent and heterogenous disease of horses. The milder forms of equine asthma are considered transient in most cases^26^ in contrast with the incurable severe form (“heaves”) which affects approximately 14% of horses in temperate climates^27^. Severe equine asthma is characterized by variable airway obstruction, bronchial remodeling and inflammation^28^ and it occurs in genetically susceptible subjects in which periods of exacerbation and remission alternate concurring with environmental antigenic exposure, similarly to the fluctuating symptomatology in human asthmatics. This disease is well suited for the evaluation of azithromycin’s clinical effects as neutrophils are the predominant leukocyte in the airways during exacerbation and corticosteroids do not resolve airway neutrophilia despite being more effective to relieve airway obstruction^29^. Also, the large size of the horse allows sequential sampling of lung tissues by thoracoscopy for the assessment of peripheral airway remodeling^28^. Inhaled corticosteroids (ICS), combined or not with bronchodilators, only partially reduce the ASM mass in equine asthma^30,31^. Whether bronchial remodeling can be further improved with medication targeting neutrophilic inflammation and ASM biology is unknown. The hypothesis of this study was that the administration of azithromycin would potentiate the effects of ICS on lung function, airway inflammation and bronchial remodeling in asthma. The primary outcome was the evaluation of airway remodeling in central and peripheral airways in response to treatment. Other objectives were to characterize the effects of adding azithromycin to ICS on measurements of airway obstruction, luminal neutrophilia and neutrophilic mediators.

## Materials and methods

### Study protocol

All experimental procedures were performed in accordance with the Canadian Council for Animal Care guidelines and were approved by the Animal Care Committee of the Faculty of Veterinary Medicine of the Université de Montréal on October 11, 2016 (Protocol # Rech-1324). This manuscript follows the recommendations of the ARRIVE guidelines.

Twelve horses with severe asthma were studied in this randomized blind controlled clinical trial. The group allocation was based on resistance ranking values. In the fluticasone and the fluticasone + azithromycin (F + A) groups respectively, horses weighed 506 ± 35 kg and 481 ± 18 kg and were aged 13.6 ± 1.4 and 13.8 ± 2.5 years (p-NS). There were four females and one castrated male in the fluticasone group and two females and four castrated males in F + A group. For five months, all horses were treated with fluticasone (GlaxoSmithKline Inc., Mississauga, ON, Canada; 2500 μg q12h by inhalation) and six of these horses also received azithromycin orally (Pharmascience, Montreal, QC, Canada and Sandoz, Boucherville, QC, Canada; 10 mg/kg q24h for five days, then q48h). Because of poor clinical response based on lung function data, the fluticasone dose was increased to 3000 µg q12h five weeks after initiation of therapy in two horses (one horse in each group) for ethical reasons. One horse in the fluticasone-treated group was excluded as it developed a persistent pneumothorax after the first thoracoscopy, which resolved after specific treatment.

### Pulmonary function tests

Lung function measurements (standard lung mechanics and oscillometry) were performed at baseline, at week (W) two, W4, W6, W8, W12, W16 and W20 (details in online Supplement). At the end of the study, lung function was assessed after the administration of an anticholinergic bronchodilator (N-butylscopolammonium bromide, 0.3 mg/kg IV; Boehringer Ingelheim, Burlington, ON, Canada) to determine if residual bronchospasm persisted^32^.

### Bronchoscopy and thoracoscopy

Bronchoscopies were performed at baseline, on W2, W4, W6, W8, W12, W16 and W20 to sample bronchoalveolar lavage fluid (BALF), endobronchial biopsies (EBBs) and to record endobronchial ultrasound (EBUS; baseline, W8, W20). The BALF samples were processed for cytology and gene expression studies and the EBBs for histomorphometry and gene expression analysis. A blinded pathologist evaluated several components of the epithelium, submucosa and ASM to attribute a remodeling score to each biopsy^33^ and the thickness of the extracellular matrix (ECM; region between the basal membrane and the ASM) was measured. On EBUS images, the area of the second layer (L2; corresponding to 70% of ASM^34^), and the internal perimeter (Pi) were measured and L2 area/Pi^2^ calculated. Biopsies of the caudo-dorsal region of the lungs were obtained at baseline, at W8 and at W20 by thoracoscopy to evaluate the ASM and ECM areas in peripheral airways. Detailed bronchoscopy and thoracoscopy procedures are described in the online Supplement.

### Peripheral polymorphonuclear isolation and high-performance liquid chromatography-mass spectrometry

Plasma azithromycin concentration was measured by high-performance liquid chromatography-mass spectrometry at W2, W8 and W20. Polymorphonuclear cells (PMNs), mostly composed of neutrophils, were isolated by density gradient centrifugation at baseline, W8 and W20 for gene expression studies and for determination of intracellular azithromycin concentration at W20 (details in online Supplement).

### Transcriptomic analysis

Messenger ribonucleic acid (mRNA) expression of interleukin-1β (IL-1β), interleukin-8 (CXCL-8) and glyceraldehyde 3-phosphate dehydrogenase (GAPDH; Table 1) was assessed by RT-qPCR from BALF cells, EBBs and PMNs at baseline, W8 and W20. The absolute quantitative gene expression was normalized to the expression level of the reference gene GAPDH. The ratio of the smooth muscle myosin heavy chain (SMMHC) ( +) insert, which is implicated in faster rate of muscle contraction^35^, to total SMMHC gene expression was evaluated in EBBs^36^ (details in online Supplement).

**Table 1:** Sequences of primer pairs used for quantitative PCR analysis.

Genes	Sequences
EC C-X-C motif chemokine ligand 8	F: 5′- CAAGCTGGCTGTTGCTCTCTTG -3′
	R: 5′- CTCAGTCCTCTTTAGAAACGCC -3′
EC interleukin-1β	F: 5′- AGTGGTGTTCTGCATGAGCTTTG -3’
	R: 5′- GTATTGGGGTCTACTGTCTCCA -3’
EC glyceraldehyde-3-phosphate dehydrogenase	F: 5′- CACCCAGAAGACCGTGGATG -3′
	R: 5′- TGCCAGTGAGCTTCCCATTC -3′
EC smooth muscle myosin heavy chain	F: 5′- TGAACAAGGCCCTGGACAAGAC -3′
	R: 5′- TGCAGCTTCTCGTTGGTGTAGT -3′
EC smooth muscle myosin heavy chain (+) insert isoform	F: 5′- ATTCTATGCACAGGCGAGTCTGGA -3′
	R: 5′- GTAGGCAAGAGGTGGGCCTTG -3′
EC, Equus caballus	

### Statistical analyses

Power calculations (G*Power 3.1.9.4, Kiel, Germany) indicated that six horses per group were required to detect a two-fold difference in the reduction of the ASM mass between groups (alpha of 0.05 and power of 80%) considering the expected improvement of 30% by fluticasone^30,31^. Two-way repeated measures ANOVA followed by Dunnett’s or Sidak’s multiple comparison tests were performed as appropriate using GraphPad Prism version 8.4.3 for Windows (GraphPad Software, San Diego, CA, USA). Normality was assessed with Shapiro–Wilk tests and visual analysis of QQ-plot, and most data sets respected normality. As log transformations did not substantially modify the results when raw data were not normally distributed, the latter were used for all analysis. In the few instances where data were missing, mixed-effect models were used and indicated as such in the figure legends. Associations between lung function, inflammation and remodeling data were explored with Pearson correlations. Descriptive data are expressed as means and standard error of the mean, or with the median and interquartile range when not normally distributed. Results were considered statistically significant at p ≤ 0.05.

## Results

### Lung function

All horses were in exacerbation of the disease prior to the beginning of the study (resistance [R_L_] > 1 cm H_2_O/L/s and elastance [E_L_] > 1 cm H_2_O/L).

**Figure 1:**
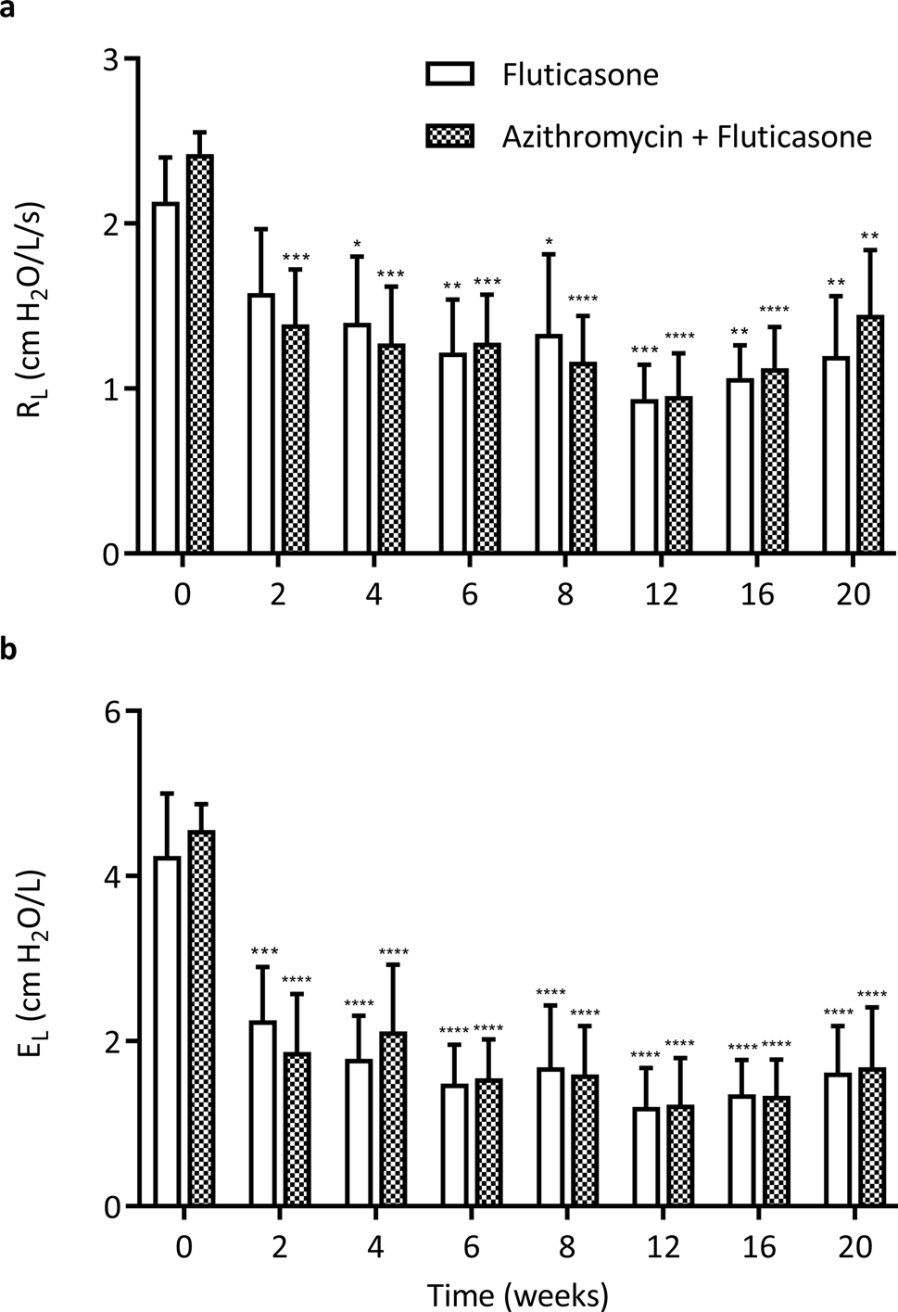
Lung function measured by standard respiratory mechanics. Values of pulmonary resistance (R_L_; **a**) and pulmonary elastance (E_L_; **b**) (mean and standard error of the mean). There was a significant main time effect but not group difference with the two-way ANOVA (p < 0.0001) for both R_L_ and E_L_. *p < 0.05, **p < 0.01, ***p < 0.001, ****p < 0.0001 compared to baseline values with Dunnett’s multiple comparison tests.

**Figure 2:**
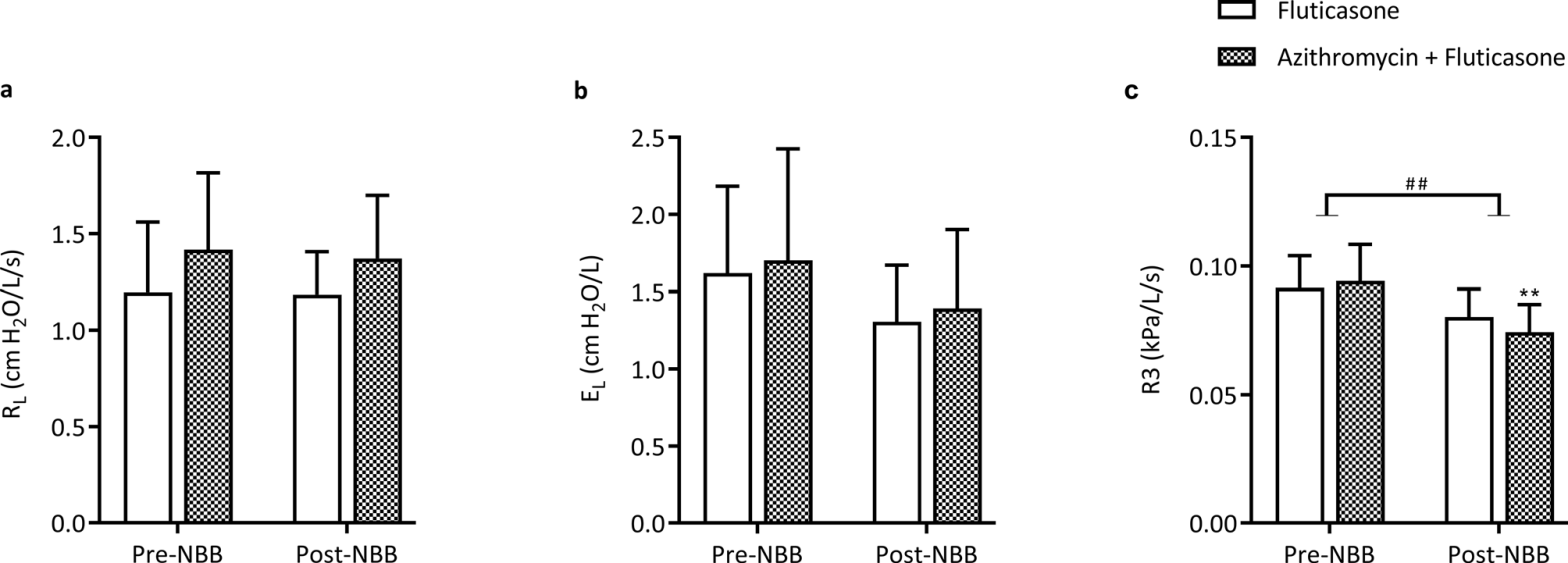
Bronchodilator test. Values of pulmonary resistance (R_L_; **a**), elastance (E_L_; **b**) and resistance at 3 Hz (R3; **c**) before and after administration of N-butylscopolammonium bromide (NBB) (mean and standard error of the mean). **p < 0.01 compared to pre-NBB with Sidak’s multiple comparison tests and ^##^p < 0.001 for the main bronchodilator effect.

Lung resistance and elastance decreased over time (p < 0.0001; Fig. 1) similarly between the two groups. However, the R_L_ and/or E_L_ values remained above normal (> 1 cm H_2_O/L/s and > 1 cm H_2_O/L respectively) in 8/11 horses at the end of the study. The lung function measured by oscillometry (Supplementary Fig. 1) showed similar results; the R5/R10 ratio, an indicator of the frequency dependence of the respiratory system resistance, decreased over time (p < 0.0001) with no difference between groups. The resistance at 3 Hz (R3), R3 during expiration, the reactance at 3 Hz (X3) and X3 during expiration improved similarly over time (p < 0.02; data not shown) while R3 and X3 during inspiration remained unchanged. The administration of N-butylscopolammonium bromide did not modify resistance, elastance and X3. However, R3 decreased significantly in response to the anti-cholinergic bronchodilator (p = 0.002; Fig. 2), with no difference between groups. Overall, the bronchodilation test showed minimal bronchospasm at the end of the study.

### Airway inflammation

**Figure 3:**
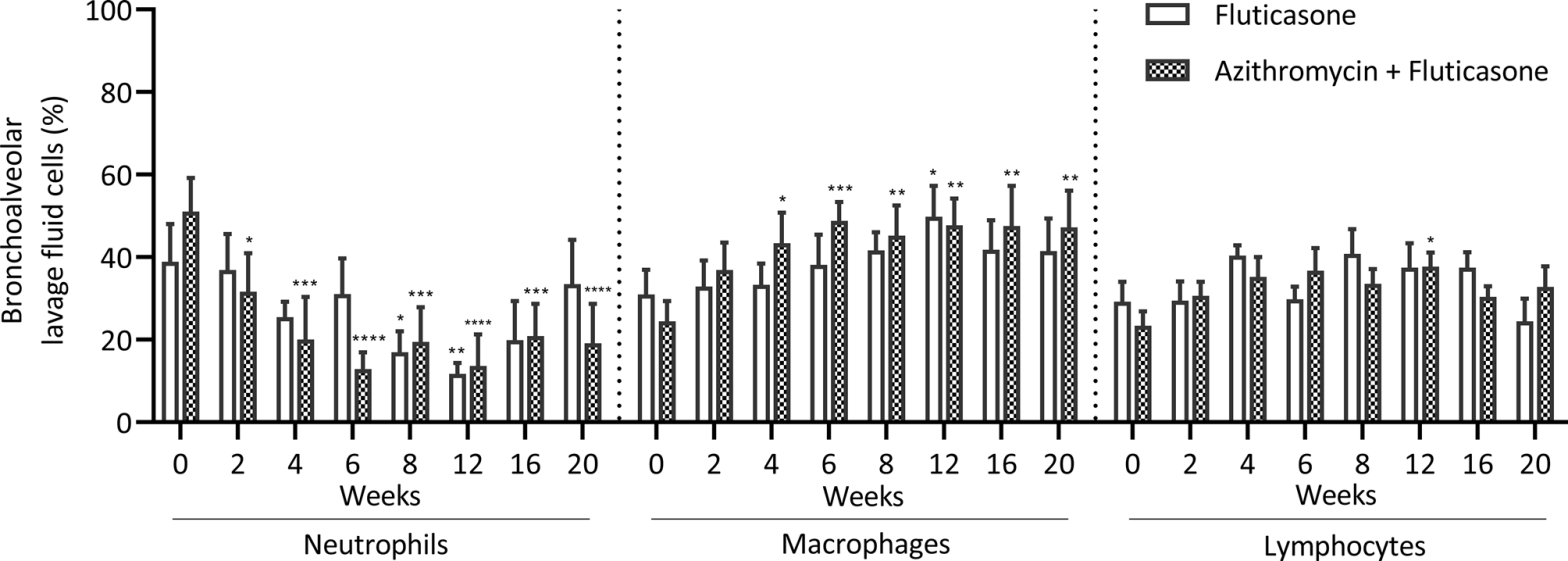
Bronchoalveolar lavage fluid cytology (mean and standard error of the mean). There was a significant time main effect but no group difference with the two-way ANOVA for neutrophils (p < 0.0001), macrophages (p = 0.0004) and lymphocytes (p = 0.01). *p < 0.05, **p < 0.01, ***p < 0.001, ****p < 0.0001 compared to baseline values with Dunnett’s multiple comparison tests.

A significant reduction of airway neutrophilia was observed over time (p < 0.0001) with a difference from W2 to W20 compared to baseline in the F + A group and at W8 and W12 in the fluticasone group (Fig. 3). The proportional change compared to the baseline value was also analyzed due to the large variation in airway neutrophilia at the beginning of the study. The proportional improvement of airway neutrophilia was significantly larger in the F + A group (group difference; p = 0.02). At the end of the study, normalization of airway neutrophilia (neutrophils ≤ 10%) occurred in 4/6 horses in the F + A group and 0/5 in the fluticasone group. As the proportion of neutrophils decreased over time, concurrent increases in the percentage of macrophages (p = 0.0004) and of lymphocytes (p = 0.01) were observed in the BALF, without group differences. The proportions of mast cells and eosinophils were low and did not vary during the study (data not shown). The percentage of BALF volume recovered increased over time (p = 0.0002). The BALF total cell counts did not vary over time or between groups, but there was an interaction between time and groups (p = 0.009; Supplementary table 1).

### Transcriptomic analysis

The gene expression of GAPDH did not vary over time or between groups in BALF cells, PMNs and EBBs, and was therefore considered an appropriate reference gene. There was no group difference for all the genes examined. In BALF cells (Supplementary Fig. 2), the expression of IL-1β decreased over time (p = 0.003), while CXCL-8 gene expression did not vary significantly (p = 0.08). The viability and purity of PMNs (neutrophils) were respectively 98% ± 0.3% and 99% ± 0.1% and were considered adequate for assessment. In those cells, the gene expression of CXCL-8 (p = 0.006), but not of IL-1β (p = 0.09), decreased over time (Supplementary Fig. 3). In EBBs, the gene expression of IL-1β (p = 0.001) and CXCL-8 (p = 0.01), and the ratio of the (+) insert isoform on total SMMHC (p = 0.002) were reduced over time (Supplementary Fig. 4).

### Histomorphometry of central and peripheral airways

**Figure 4:**
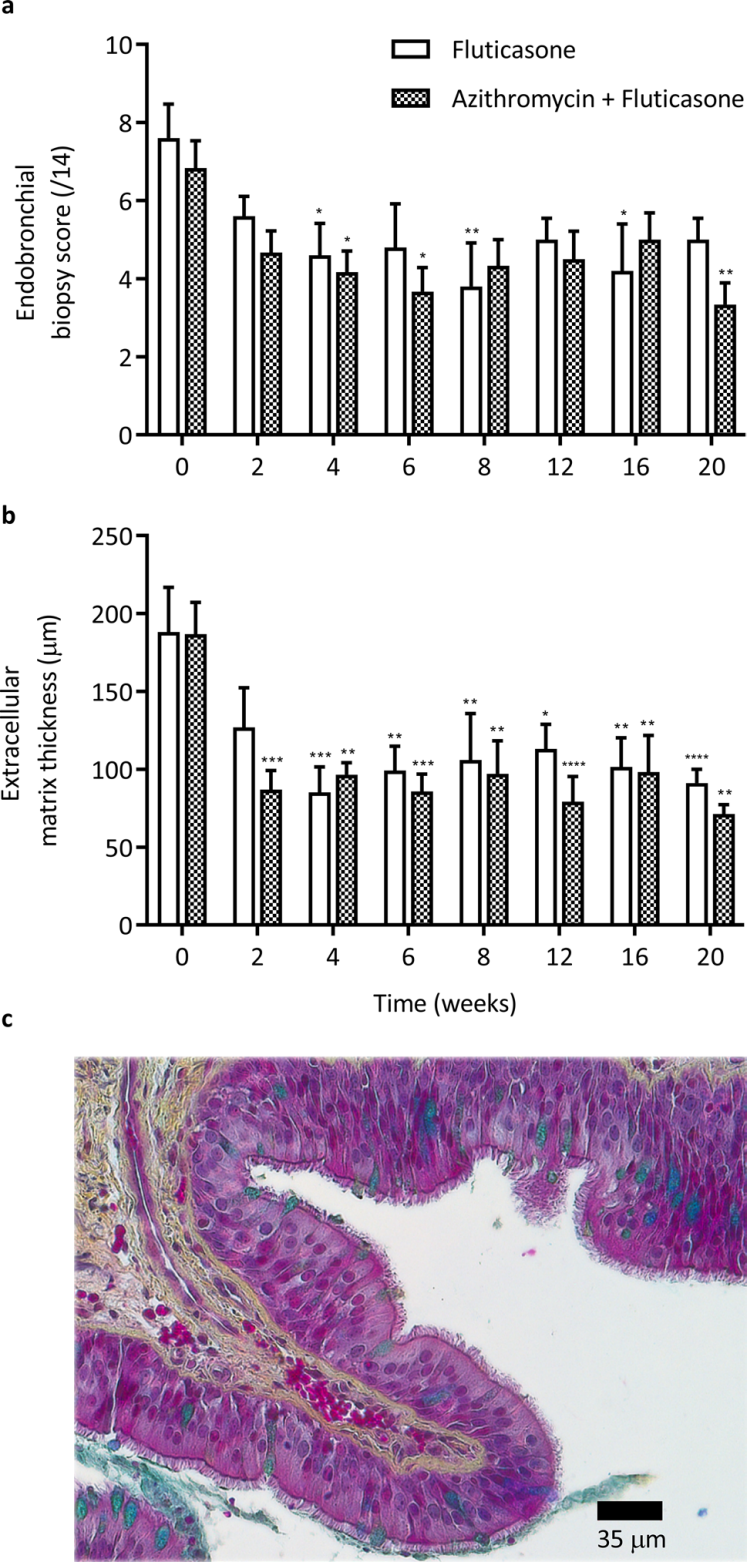
Endobronchial biopsies. Histological score (**a**) and extracellular matrix thickness (**b**) of the endobronchial biopsies (mean and standard error of the mean). There was a significant time main effect but no group difference with the two-way ANOVA for the score (p = 0.001) and the extracellular matrix thickness (p < 0.0001). *p < 0.05, **p < 0.01, ***p < 0.001, ****p < 0.0001 compared to baseline values with Dunnett’s multiple comparison tests. c) Epithelium from a central airway stained with Russell-Movat-pentachrome.

The quality score of the EBBs was 3.8 ± 0.07 (good to optimal quality) and did not vary over time or between groups. In central airways (Fig. 4C), the score reflecting severity of asthma-related architectural changes decreased over time (p = 0.001) in both groups (Fig. 4A), as did the thickness of the ECM (p < 0.0001; Fig. 4B; median decrease of 67.6% (44.1–71.3) and 51.3% (29.8–63.0) in the F + A group and fluticasone group, respectively at W20 compared to baseline).

**Figure 5:**
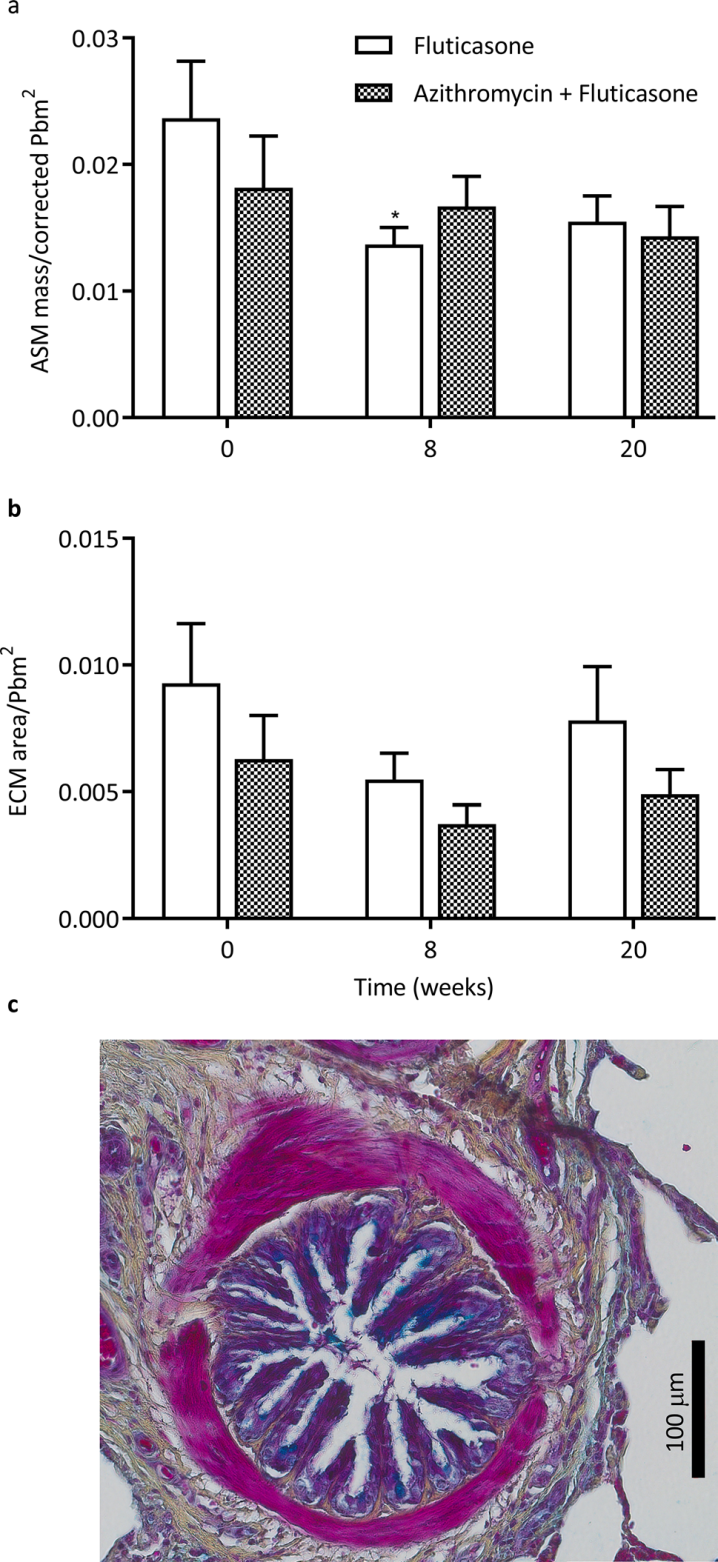
Peripheral bronchial remodeling. (**a**) Airway smooth muscle mass (ASM) corrected for the perimeter of basal membrane where the muscle was located (corrected Pbm^2^) (mean and standard error of the mean). There was a significant time main effect but not group difference with the two-way ANOVA (p = 0.05). (**b**) Extracellular matrix area (ECM) corrected for the length of the basal membrane (Pbm^2^). *p < 0.05 compared to baseline values with Dunnett’s multiple comparison tests. c) Peripheral bronchi stained with Russell-Movat-pentachrome.

Peripheral airway remodeling was evaluated in 7.2 ± 0.5 (range 2–19) airways per horse per time point (Fig. 5C). The peripheral ASM mass decreased over time (p = 0.05; Fig. 5A) with no difference between groups. This represented a median decrease of 21.7% (− 110.8–40.7) and 24.7% (-31.1–47.6) at W8 and W20 for the F + A group, and of 38.2% (17.1–52.8) and 34.3% (7.6–48.0) at W8 and W20 for the fluticasone group. The peripheral ECM area did not vary over time or between treatment groups (Fig. 5B).

### Endobronchial ultrasound

**Figure 6:**
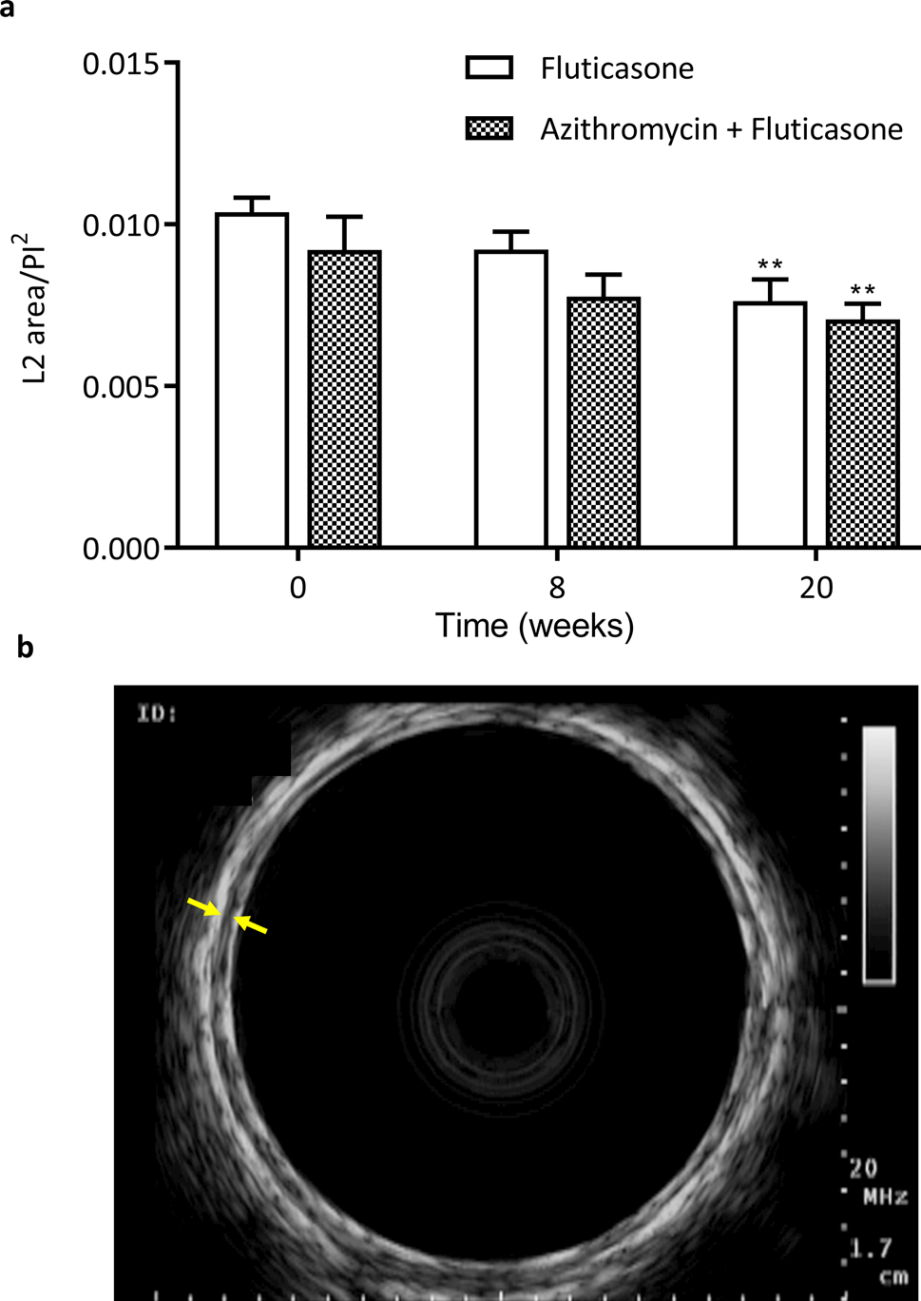
Endobronchial ultrasound. (**a**) Area of the second layer (L2) corrected by the internal perimeter (PI) (mean and standard error of the mean). There was a significant time main effect but no group difference with the two-way ANOVA (p = 0.0002). **p < 0.01 compared to baseline values with Dunnett’s multiple comparison tests. (**b**) Central airway obtained with endobronchial ultrasound. The distance between the yellow arrows illustrates the thickness of L2.

Central airway remodeling was assessed in 6.3 ± 0.3 (range 4–9) bronchi per horse per time point. The ASM mass (L2/PI^2^) in central airways decreased significantly over time (p = 0.0002), similarly in each group (Fig. 6; mean decrease of 13.4 ± 7.4% and 21.1 ± 5.5% at W8 and W20 for the F + A group, and of 10.1 ± 8.1% and 25.7 ± 8.2% at W8 and W20 for the fluticasone group).

### Azithromycin concentration

In the six horses receiving azithromycin, the plasma concentration at W2, W8 and W20 was 85.5 ± 29.6, 84.8 ± 20.4 and 86.9 ± 22.1 ng/ml. Mean intracellular concentration of azithromycin at W20 was 10,758 ± 3,224 ng/ml of PMNs. On average, intracellular concentration of azithromycin was 158 times higher than in plasma at W20.

### Associations between clinicopathological data and remodeling

At baseline, airway neutrophilia correlated with lung function data evaluated with oscillometry (significant correlations between neutrophilia and R3 [r = 0.75, p = 0.01] and X3 [r = − 0.75, p = 0.01] during inspiration). The BALF neutrophil percentage at baseline was also associated with poorer lung function improvement at W20 (correlations between BALF neutrophilia and R_L_ W20/R_L_ baseline [r = 0.65, p = 0.03] and E_L_ W20/E_L_ baseline [r = 0.67, p = 0.02]). At baseline, the gene expression of IL-1β in PMNs correlated with the R5/R10 ratio (r = 0.80, p = 0.003), while its expression in BALF cells correlated with airway neutrophilia (r = 0.71, p = 0.02). At the beginning of the study, the L2/PI^2^ area measured with EBUS correlated with the ASM mass in peripheral airways (r = 0.61, p = 0.05). Pulmonary resistance (r = − 0.69, p = 0.02) and elastance (r = − 0.74, p = 0.009) were negatively correlated with the ECM area in peripheral airways at baseline.

### Adverse effects

The horses receiving azithromycin displayed softer manure for the duration of the study, as previously reported in horses^37,38^ and concordant with diarrhea being a frequent side effect in humans^12^. At the end of the study, all horses had localized alopecia around the nares, where the inhaler device was positioned on the skin. Bacteriological and fungal cultures did not reveal infectious pathogens. Histologic evaluation of skin biopsies showed follicular and epidermal atrophy compatible with corticosteroid topical effects. The results of the complete blood counts and serum chemistry were within the expected values for horses affected by severe asthma at baseline and at the end of the study except for two mares that had a mild increase in liver enzymes at W20 (fluticasone group), without any related clinical signs.

## Discussion

The administration of azithromycin has beneficial effects on some features of asthma, however, whether these favorable properties extend to the airway architectural changes has not been assessed in natural models of asthma. Combining azithromycin to ICS reduced airway neutrophilia further than the sole administration of fluticasone in severe equine asthma. Yet, the improvement of lung function and bronchial remodeling obtained with fluticasone were not potentiated by the macrolide, refuting the initial hypothesis.

Macrolides have been proposed as an add-on medication in patients with neutrophilic inflammation when symptoms are not adequately relieved by standard therapy^39^. However, conclusions from meta-analysis are conflicting regarding their effects on lung function^40–42^, and it is unclear how these drugs modulate clinical signs and exacerbation frequency in asthma. The results of the current study are consistent with reports that did not observe an improvement in respiratory mechanics with add-on macrolide administration^12,13,43^. Furthermore, the persistence of abnormal lung function in 8/11 horses at the end of the study, along with the presence of some residual bronchoconstriction, suggest a degree of corticosteroid resistance that was not reversed by the addition of azithromycin. Interestingly, the remaining bronchospasm was not detected by standard respiratory mechanics, but only by oscillometry, which supports the latter’s sensitivity for the detection of bronchodilation and poor asthma control^44^.

The negative clinical outcomes associated with the presence of neutrophils in asthma (including acute exacerbation^7^ and steroid resistance^9^), justify the search of therapies targeting this leukocyte. Macrolides decreased airway neutrophilia in some^23,41,43^, but not in all asthma reports^12^ and they are possibly more useful in face of neutrophilic inflammation^13^, although again this is not a consistent finding^12^. In the present study, the greater reduction of airway neutrophilia in the group receiving azithromycin was not accompanied by enhanced effects on remodeling and lung function, questioning the role of this leukocyte, and its different subsets and by-products, in the disease. However, it is possible that a longer control of inflammation is required to reverse bronchial remodeling.

Interleukin-1β and CXCL-8 are both secreted by neutrophils and are potent activators of these cells. The pro-inflammatory cytokine IL-1β is associated with decreased lung function, poor asthma control and requirement for high doses of ICS^10,45^, and its sputum gene expression and protein content are elevated in neutrophilic asthma^45,46^. The gene expression of IL-1β in BALF decreased after a short-term azithromycin treatment in severe equine asthma^38^. However, azithromycin did not enhance the fluticasone-induced reduction of IL-1β in BALF cells and EBBs in the current study. There is little information on the impact of glucocorticoids on IL-1β expression in human asthma and data in animal models are conflicting^45,47,48^. In contrast to the current results, others reported an increased expression of IL-1β in the sputum from patients with stable persistent asthma after one month of fluticasone treatment^46^. Species difference and varying degree of asthma severity could have contributed to those discordant results. The chemokine CXCL-8 is also elevated in neutrophilic asthma^49^. Clarithromycin decreased CXCL-8 protein content in sputum from patients with a severe refractory phenotype^43^ and a short-term azithromycin treatment reduced its expression in BALF cells from horses with severe asthma^38^. Although a significant decrease in CXCL-8 gene expression in PMNs and in EBBs occurred over time in the current study, there was no potentiation by azithromycin.

Despite airway remodeling, especially the increased ASM mass, being a major contributor to airway obstruction in asthma, it is not a consistent consideration in clinical decisions related to therapeutic interventions^50^. This study was performed to investigate whether modulation of ASM biology by macrolides contribute to their clinical efficacy. Indeed, azithromycin induces autophagy in human bronchial ASM^15^ and relaxes pre-contracted human bronchial muscle strips^17^. In vivo, macrolide administration decreases ASM mass in rodents with an induced asthma-like disease^18–21^ and in healthy mice^51^. However, these studies have assessed the effects of high doses (≥ 25 mg/kg azithromycin) while asthmatic patients usually receive much lower dosages (≤ 10 mg/kg)^12,13^. In the present clinical trial, fluticasone reduced the ASM mass to a similar extent than previous results in this equine model^30,31^. However, this parameter was not further improved by the administration of azithromycin at a dose representative of current asthma therapy. Similarly, the macrolide did not modify the ASM mass in the central airways. The L2/PI^2^ area measured with EBUS decreased by ≈25% after five months of treatment in both groups, an improvement similar to previously reported with the combination of fluticasone and salmeterol^31^ and comparable to what was observed for peripheral airways. The multivariate score used to evaluate histological lesions in the epithelium, submucosa and ASM in central airways^33^ also showed that therapy lessened the architectural alterations in this asthma model, but without a group difference. Combined, these results contrast with mathematical models predicting a similar effectiveness of azithromycin and bronchial thermoplasty for the treatment of asthma via a reduction of the ASM mass^51^.

The increased gene expression of the SMMHC (+) insert isoform in ASM from human^35^ and equine^36^ central asthmatic bronchi could be implicated in airway hyperresponsiveness because it induces a faster rate of muscle contraction. In the current study, the mRNA expression of the (+) insert isoform to total SMMHC ratio was concordant to previous results in asthmatic horses^36^, but the improvement (≈ two-fold reduction) obtained with ICS was not potentiated by azithromycin. Based on this finding, perhaps the decreased airway hyperresponsiveness observed with macrolides in some studies^22,23^ is not related to the modulation of the SMMHC (+) insert isoform and ASM remodeling.

This study revealed several relevant associations between histomorphometry and clinical data. First, the correlation between the ASM mass in small bronchi and the L2/PI^2^ area in the central airways was confirmed^31^ and supports the utility of EBUS as a surrogate marker of peripheral remodeling. Surprisingly, negative correlations between lung function data and the ECM area in peripheral biopsies were observed at baseline, indicating that subjects with higher deposition of ECM had less airway obstruction. A potential explanation would be that the increased stiffness provided by these tissues could partially prevent the ASM-induced bronchoconstriction or that the increased matrix deposition may enhance the forces of interdependence between parenchyma and airway. This study also identified associations between BALF neutrophilia and parameters of lung function measured by oscillometry, which has been previously described in human asthma^52^ and suggests that this technique may be useful to detect peripheral dysfunction related to inflammation-mediated bronchoconstriction. Finally, the poorer improvement of lung function in subjects with higher airway neutrophilia at baseline is consistent with the corticosteroid resistance reported in human asthmatics with this type of inflammation^9^.

A limitation of this study was the low number of horses evaluated, which could have precluded the detection of differences between groups. However, there was no trend suggesting that azithromycin would be useful in reversing bronchial remodeling in this natural model of asthma. Severe equine asthma is a condition also known as heaves, recurrent airway obstruction and chronic obstructive pulmonary disease (COPD). The use of the latter has been abandoned as the disease in horses do not share etiological or histopathological findings with COPD, in contrast with the comparable bronchial remodeling and response to therapy of asthma in humans^28^. Despite these similitudes, it remains possible that macrolides have different effects in human subjects than observed in the current study with horses. Furthermore, the inclusion of an untreated control group would have been preferable to assess the impact of external factors, but it would have been unethical as no improvement of lung function is expected when horses are kept in an antigenic environment.

As specific pathogens can play a role in human asthmatic exacerbations, macrolide benefits might originate from their antimicrobial properties. Thus, assessing the influence of antibiotic treatment on the microbiome and particularly antimicrobial resistance is important. Indeed, azithromycin reduced bacterial density and increased macrolide and non-macrolide resistance genes from sputum samples of asthmatic subjects^53^. Therefore, another limitation of this study was the lack of microbiological assessment in the airways. On the other hand, viral and bacterial infections are not contributing to exacerbations in severe equine asthma, and azithromycin administration does not improve lung function or modify tracheal bacterial counts in this disease^38^.

Of note, the plasma and PMN intracellular concentrations of azithromycin obtained in asthmatic horses were comparable to those found in humans^54^ and were concordant with previous pharmacokinetic results in the equine species^37^. Therefore, the poor clinical response to azithromycin in this trial is unlikely related to insufficient drug concentrations.

In conclusion, this study indicates that the positive clinical outcomes observed with macrolides are perhaps not due to modulation of bronchial remodeling. Understanding how azithromycin is clinically useful is still pertinent to further uncover asthma pathophysiology, to justify its use in severe cases and ultimately to guide the development of non-antimicrobial macrolides.

## Supplementary Information

Supplementary Information.

## Data Availability

The datasets generated in the current study are available in the Dataverse UdeM repository, [10.5683/SP2/QM67NC].
