# Supplementary material for: Effects of azithromycin on bronchial remodeling in the natural model of severe neutrophilic asthma in horses
Source: Sci Rep. 2022 Jan 10;12:446. doi: 10.1038/s41598-021-03955-9 (PMC8748876; doi:10.1038/s41598-021-03955-9)
Supplement: Supplementary file 1 — Supplementary Information. [file 41598_2021_3955_MOESM1_ESM.pdf]

Supplement to "Effects of azithromycin on bronchial remodeling in the natural model of severe neutrophilic asthma in horses"

Sophie Mainguy-Seers, Roxane Boivin, Sheila Pourali Dogaheh, Francis Beaudry, Pierre Hélie, Alvaro G. Bonilla, James G. Martin, Jean-Pierre Lavoie

MATERIALS AND METHODS

The protocol of this study was designed and described in the grant application for the Canadian Institutes of Health Research. The protocol was not registered.

Animals

Horses were donated to the research herd because of severe asthma. The diagnosis was confirmed based on history of chronic respiratory signs, pulmonary function test results and bronchoalveolar lavage fluid (BALF) cytology[1]. During antigenic exposure (stabling and hay feeding), these horses had > 25% neutrophils on BALF cytology (normal ≤ 10%) and a pulmonary resistance (R_L) and elastance (E_L) > 1 cm H_2O/L/s and > 1 cm H_2O/L, respectively. Physical examination, complete blood count, serum biochemistry and thoracic radiographs were assessed to rule out other respiratory conditions. To induce and maintain clinical exacerbation, horses were stabled (wood shaving bedding) and fed dry hay starting one month before and for the duration of the trial. Daily outdoor turnout times were predetermined based on the individual clinical severity during asthma exacerbation. The management of each horse remained the same throughout the study period. Horses were conditioned to wear a mask, to stand in a stock and to receive inhaled medication delivered with the AeroHippus inhaler mask (Trudell Medical International, London, ON, Canada). Horses were monitored twice daily for the duration of the study. Human endpoints included anorexia, abnormal fecal output, hyperthermia, colic, respiratory distress, or any other medical conditions. If the condition or its treatment interfered with the study, the horse would be excluded. Housing and manipulations occurred at the research farm, except for the thoracoscopies which were performed at the Centre Hospitalier Universitaire Vétérinaire of the Université de Montréal. Each horse was ranked based on the baseline resistance value (horse with the highest resistance in group A, horse with the second highest resistance in group B, horse with the third highest resistance in group A and so on), then groups were randomly allocated by coin toss.

Pulmonary function tests

The standard respiratory mechanics were performed in unsedated standing horses. Briefly, a polyethylene catheter with an air-filled (5 ml) balloon at its tip was inserted in the distal third of the esophagus to obtain a measurement of the pleural pressure (P_L). Flow rates were measured during a two-minute period with a heated pneumotachograph and a differential pressure transducer fitted to a mask placed over the horse's nose. The head was positioned to minimize upper airway resistance during measurements[2]. The commercial software (Flexiware 7.6, SCIREQ, Montreal, QC, Canada) allowed electronic integration of the flow to obtain volume, and together with the pressure signals to obtain values of pulmonary resistance (R_L) and elastance (E_L) by applying multiple linear regression to the equation for the single compartment model of the lung ($P_L = E_L V + R_L \dot{V} + K$); *V* is the volume, $\dot{V}$ the airflow and *K* the transpulmonary end-expiratory pressure. All valid breaths obtained over the two-minute period were averaged for analysis.

Lung function was also measured with oscillometry as previously described[3] as it is possibly more sensitive than standard lung function measurements for detecting airway obstruction[4]. The Equine MasterScreen impulse oscillometry system (Jaeger GmbH, Würzburg, Germany) was calibrated before each use. Multi-frequency impulses generated by a loudspeaker were superimposed on the tidal breathing by an airtight mask placed over the horse's nose. The pressure-flow signal response of the respiratory system was measured by a pressure transducer connected to a pneumotachograph attached to the front of the facemask. Three recordings of 30 seconds duration were acquired. Analysis were performed with LabManager (version 4.53, Jaeger, Würzburg, Germany) and FAMOS (IMC, Meβsysteme, Berlin, Germany) using Fast-Fourier transform. Inspiratory (insp), expiratory (exp) and resistance (R3) and reactance (X3) of the respiratory system at 3 Hz were analyzed. The ratio of resistance at 5 Hz and 10 Hz (R5/R10) was used as an indicator of the frequency dependence of the respiratory system resistance.

Bronchoscopy and bronchoalveolar lavage (BAL)

Endoscopy was performed in sedated horses (detomidine [Dormosedan, Zoetis, Kirkland, QC, Canada; 0.01 mg/kg, IV] and butorphanol [Torbugesic, Zoetis; 0.02 mg/kg, IV]) with a 1.6 meter videoendoscope (12.8-mm external diameter; Evis Exera II CV-180, Olympus Canada, Richmond Hill, ON, Canada). The bronchoalveolar lavages (BAL) were performed as previously described [5] by instilling 250 ml of warm sterile isotonic saline into a main bronchus through the videoendoscope and immediately aspirated with a suction pump, and this was repeated once in the same airway. The samples were kept on ice until processing within 90 minutes. Cytocentrifuged preparations of bronchoalveolar lavage fluid (BALF) were stained with a modified Wright–Giemsa solution (Diff-Quik, Fisher Scientific, Waltham, Massachusetts, USA). Differential leukocyte counts were performed blindly from 400 cells. In the equine species, BALF

differential cell count is provided instead of total counts for each leukocyte as the BALF volume recovered is dependent on the degree of airway obstruction[6]. BALF was centrifuged at 500 x *g* for five minutes, then the pelleted cells were washed twice in PBS, and 10 million cells were re-suspended in Trizol reagent (Invitrogen, Carlsbad, CA, USA) and kept at −80°C until RNA extraction.

Endobronchial ultrasound

Endobronchial ultrasound was performed as previously reported[7] with a 20 MHz radial miniature probe (UM-BS20-26R, Olympus Canada) with the compatible balloon-ended sheath (MAJ-643R, Olympus Canada). The images with the highest quality were analyzed by a blinded investigator (at least 3 images/airway)[7] with Image J (version 1.52a, NIH, Bethesda, USA).

Endobronchial biopsies

Endobronchial biopsies were collected after BAL and EBUS, as previously described[8]. Topical 0.5% lidocaine hydrochloride (Vétoquinol N.-A. Inc., Lavaltrie, QC, Canada) was used to anesthetize bronchial carinae and endobronchial biopsies were harvested using smooth oval forceps (Standard Fenestrated and Smooth, 2.3 m, Olympus Medical Systems, Tokyo, Japan) from different branching sites as previously reported[8]. The biopsies were mainly collected between the second and ninth bronchial bifurcations. Ten biopsies were collected at each time point. Six were fixed for 2-4 h in 10% neutral-buffered formalin and then embedded in paraffin and four were saved to preserve RNA (RNA*later*™ Stabilization Solution, Thermo Fisher Scientific, Burlington, ON, Canada). Histologic sections of 4 µm thickness were obtained from several biopsies, stained with Russell-Movat-pentachrome and digitized at 40x magnification with a Leica microscope (DCF320, Leica Microsystems, Cambridge, UK) and the Panoptiq software (ViewsIQ, version 1.4.3, Vancouver, BC, Canada). A quality score was attributed to each biopsy[8] and for each horse at each time point, the highest quality biopsy was used. The thickness of the extracellular matrix (ECM; area between the basal membrane and airway smooth muscle) was measured five times with Image J (version 1.52a, NIH) and averaged on each biopsy.

Peripheral lung biopsies

Peripheral biopsies of the caudo-dorsal region of the lungs were obtained by thoracoscopy by a board-certified surgeon in large animal surgery under standing intravenous sedation, with a bolus of detomidine [0.01 mg/kg] and butorphanol [0.02 mg/kg] being followed by a detomidine (0.08 mg/ml) continuous rate of infusion titrated to effect. Similarly to previously described[9], one portal at the dorsal aspect of the 13th or 14th intercostal space was performed for the thoracoscope and two instrument portals were then added

at the 13th and 15th intercostal space (10 cm distally to the thoracoscope portal). Pulmonary wedge biopsies were obtained with an endoscopic stapler (60-mm or 45-mm of operating length and a staple size of 4.8-mm, Endo GIA, Covidien) and were retrieved using an endoscopic specimen bag (AKYQWA-80, Disposable Endo Bags, Changzhou Ankang Medical Instruments Co, Ldt., Changzhou, Jiangsu, China). Non-steroidal anti-inflammatory drugs (phenylbutazone 200 mg/ml; McCarthy and Sons Service, Calgary, AB, Canada; 4.4 mg/kg IV q24 for 48 hours) were given to all horses and an antimicrobial (penicillin; Intervet Canada Corp., subsidiary of Merck & Co., Inc., Kirkland, QC, Canada; 22 000 IU/kg IM once) was administered to horses not receiving azithromycin directly after biopsy retrieval. Biopsies were fixed in 10% neutral-buffered formalin for 24 hours and then processed as were the endobronchial biopsies. Peripheral bronchi with a diameter <2 mm and a major to minor axis ratio ≤ 1.5 were analyzed using Image J (version 1.52a, NIH). The airway smooth muscle (ASM) and ECM areas were measured manually by tracing the external borders of each region. The ECM corresponded to the area between the basal membrane (Pmb) and ASM. The Pmb length was traced manually and used for correction attributable to variation of airway size[10].

Blood collection and peripheral polymorphonuclear isolation

Blood was collected in the morning via jugular venipuncture into K_3-EDTA tubes (Covidien, Mansfield, MA, USA). After resting for 40 minutes, plasma was isolated by centrifugation at 900 x *g* for 10 minutes at room temperature and stored at -80°C. For neutrophil isolation, the plasma-rich layer was recovered after 40 minutes of sedimentation and subjected to a density gradient centrifugation with Ficoll-PaqueTM Premium 1.084 (GE Healthcare Bio-sciences Corp., Mississauga, Ontario, Canada) from blood drawn in K_3-EDTA tubes. Erythrocytes were removed from the samples by hypotonic lysis with distilled water (Thermo Fisher Scientific). Peripheral polymorphonuclear cells (PMNs) were then washed and suspended in a buffer solution (PBS 1X, EDTA 0.5 mM [Thermo Fisher Scientific], BSA 0.2% [Sigma-Aldrich, St Louis, MO, USA]), followed by assessment of cell concentration and viability with an ADAM automatic Cell Counter (Montreal-Biotech Inc., Montreal, QC, Canada). Cytopreparations were stained with a modified Wright–Giemsa solution (Diff-Quik, Fisher Scientific) and purity was assessed by counting 400 cells. PMNs were then mixed with one millilitre of Trizol (Invitrogen) and stored at -80°C until analysis.

Azithromycin concentration

Azithromycin was extracted from plasma using protein precipitation. Two hundred µL of internal standard solution (100 ng/mL of azithromycin-d_3 in acetonitrile) was added to 50 µL of plasma samples. The sample was vortexed for five seconds and rested for 10 minutes, then centrifuged at 12 000 x *g* for 10 minutes.

The supernatant was transferred into an injection vial. The chromatography was performed using a gradient mobile phase along with a microbore column Thermo Biobasic Phenyl 50 × 1 mm, with a particle size of 5 µm. The initial mobile phase condition consisted of acetonitrile and water (both fortified with 0.1% of formic acid) at a ratio of 5:95. From 0 to 1 minute, the ratio was maintained at 5:95. From 1 to 5 minutes, a linear gradient was applied up to a ratio of 20:80 and maintained for three minutes. The mobile phase composition ratio reverted to the initial conditions and the column was allowed to re-equilibrate for seven minutes for a total run time of 15 minutes. The flow rate was fixed at 75 µL/min and 2 µL of samples were injected. A Thermo Scientific Q-Exactive Orbitrap Mass Spectrometer (San Jose, CA, USA) was interfaced with the Thermo Scientific Vanquish FLEX UHPLC system using a pneumatic assisted heated electrospray ion source. MS detection was performed in positive ion mode and operating in scan mode at high-resolution, and accurate-mass (HRAM). Nitrogen was used for sheath and auxiliary gases and they were set at 10 and 5 arbitrary units. The heated ESI probe was set to 4000 V and the ion transfer tube temperature was set to 300°C. The scan range was set to *m/z* 500-1000. Data was acquired at a resolving power of 140,000 (FWHM) using automatic gain control target of $3.0x10^6$ and maximum ion injection time of 200 msecs. Targeted drug quantification was performed at MS^1 level using specific precursor masses based on the monoisotopic masses (i.e $[M+H]^+$ ions). Quantification was performed by extracting specific precursor ions using a 5 ppm mass window. Instrument calibration was performed prior to all analysis and mass accuracy was notably below 1 ppm using Thermo Pierce calibration solution and automated instrument protocol. Azithromycin quantification was performed using peak-area ratio of azithromycin and deuterated analog azithromycin-d_3 and concentrations were determined by interpolating unknowns from the calibration curve (i.e. 2 to 1,000 ng/mL) constructed with standards prepared in plasma. The observed precision and accuracy were within +/-15%. Intracellular concentration of azithromycin in PMNs was estimated as previously described[11] at W20.

Transcriptomic analysis by qRT-PCR

For the PMN cells and the BALF pelleted cells, mRNA was extracted from Trizol reagent (Invitrogen) according to the manufacturer's instructions. For the endobronchial biopsies (two biopsies per extraction), mRNA was extracted using the RNeasy Plus Mini Kits (Qiagen, Burlington, ON, Canada) according to the manufacturer's instructions. Concentration and purity of total mRNA were assessed by spectrophotometry (Nanodrop ND1000, ThermoScientific, Wilmington, DE, USA) by measuring the 260/280 absorbance ratio. Total mRNA (0.3 µg) was reverse transcribed in duplicate using SuperScript™ III Reverse Transcriptase (Thermo Fisher Scientific, 200 U), Recombinant Ribonuclease Inhibitor (Thermo Fisher Scientific; 40 U),

one microliter of oligo(dT) (Thermo Fisher Scientific), one microliter of DTT (Thermo Fisher Scientific; 0.1M), one microliter of dNTP (Thermo Fisher Scientific; 100 mM) and 5X first-strand buffer (Thermo Fisher Scientific). QuantiTect SYBR Green PCR kits (Qiagen) were used according to the manufacturer's instructions with the Rotor-Gene RG3000 (Corbett Research, Sydney, Australia)[12]. Two microliters of cDNA were used in a final volume of 20 µL with 0.5 µM of sense and antisense primers (Thermo Fisher Scientific). For quantification of SMMHC and its (+) insert isoform, 2.75 mM $MgCl_2$ was added to the reactions. Samples were run with negative controls (reverse transcription negative and PCR negative controls) and with samples with known quantity of the evaluated gene (standard curves).

REFERENCES

1 Couetil, L. L. *et al.* Inflammatory Airway Disease of Horses--Revised Consensus Statement. *J Vet Intern Med* **30**, 503-515, doi:10.1111/jvim.13824 (2016).

2 Lavoie, J. P., Pascoe, J. R. & Kurpershoek, C. J. Effect of head and neck position on respiratory mechanics in horses sedated with xylazine. *Am. J. Vet. Res.* **53**, 1652-1657 (1992).

3 van Erck, E., Votion, D., Art, T. & Lekeux, P. Measurement of respiratory function by impulse oscillometry in horses. *Equine Vet J* **36**, 21-28, doi:10.2746/0425164044864714 (2004).

4 Van Erck, E., Votion, D., Art, T. & Lekeux, P. Qualitative and quantitative evaluation of equine respiratory mechanics by impulse oscillometry. *Equine Vet. J.* **38**, 52-58 (2006).

5 Lavoie, J. P. *et al.* Lack of clinical efficacy of a phosphodiesterase-4 inhibitor for treatment of heaves in horses. *J Vet Intern Med* **20**, 175-181, doi:10.1892/0891-6640(2006)20[175:loceoa]2.0.co;2 (2006).

6 Koblinger, K. *et al.* Bronchial collapse during bronchoalveolar lavage in horses is an indicator of lung inflammation. *Equine Vet J* **46**, 50-55, doi:10.1111/evj.12096 (2014).

7 Bullone, M., Beauchamp, G., Godbout, M., Martin, J. G. & Lavoie, J. P. Endobronchial Ultrasound Reliably Quantifies Airway Smooth Muscle Remodeling in an Equine Asthma Model. *PLoS One* **10**, e0136284, doi:10.1371/journal.pone.0136284 (2015).

8 Bullone, M., Chevigny, M., Allano, M., Martin, J. G. & Lavoie, J. P. Technical and physiological determinants of airway smooth muscle mass in endobronchial biopsy samples of asthmatic horses. *J Appl Physiol (1985)* **117**, 806-815, doi:10.1152/japplphysiol.00468.2014 (2014).

9 Lugo, J. *et al.* Safety and efficacy of a technique for thoracoscopically guided pulmonary wedge resection in horses. *Am. J. Vet. Res.* **63**, 1232-1240 (2002).

10 James, A. L., Hogg, J. C., Dunn, L. A. & Pare, P. D. The use of the internal perimeter to compare airway size and to calculate smooth muscle shortening. *Am Rev Respir Dis* **138**, 136-139, doi:10.1164/ajrccm/138.1.136 (1988).

11 Leclere, M. *et al.* Pharmacokinetics and preliminary safety evaluation of azithromycin in adult horses. *J. Vet. Pharmacol. Ther.* **35**, 541-549, doi:10.1111/j.1365-2885.2011.01351.x (2012).

12 Lavoie-Lamoureux, A. *et al.* IL-4 activates equine neutrophils and induces a mixed inflammatory cytokine expression profile with enhanced neutrophil chemotactic mediator release ex vivo. *Am. J. Physiol. Lung Cell Mol. Physiol.* **299**, L472-482, doi:10.1152/ajplung.00135.2009 (2010).

FIGURES IN SUPPLEMENT

Supplementary figure 1. Lung function measured by impulse oscillometry. a) Ratio of the resistance at 5 Hz and 10 Hz (R5/R10). b) Reactance at 3 Hz (X3) (mean and standard error of the mean). There was a significant time main effect but no group difference with the two-way ANOVA for R5/R10 and X3 values (p < 0.0001). * p < 0.05, ** p < 0.01, *** p < 0.001, **** p < 0.0001 compared to baseline values with Dunnett's multiple comparison tests.

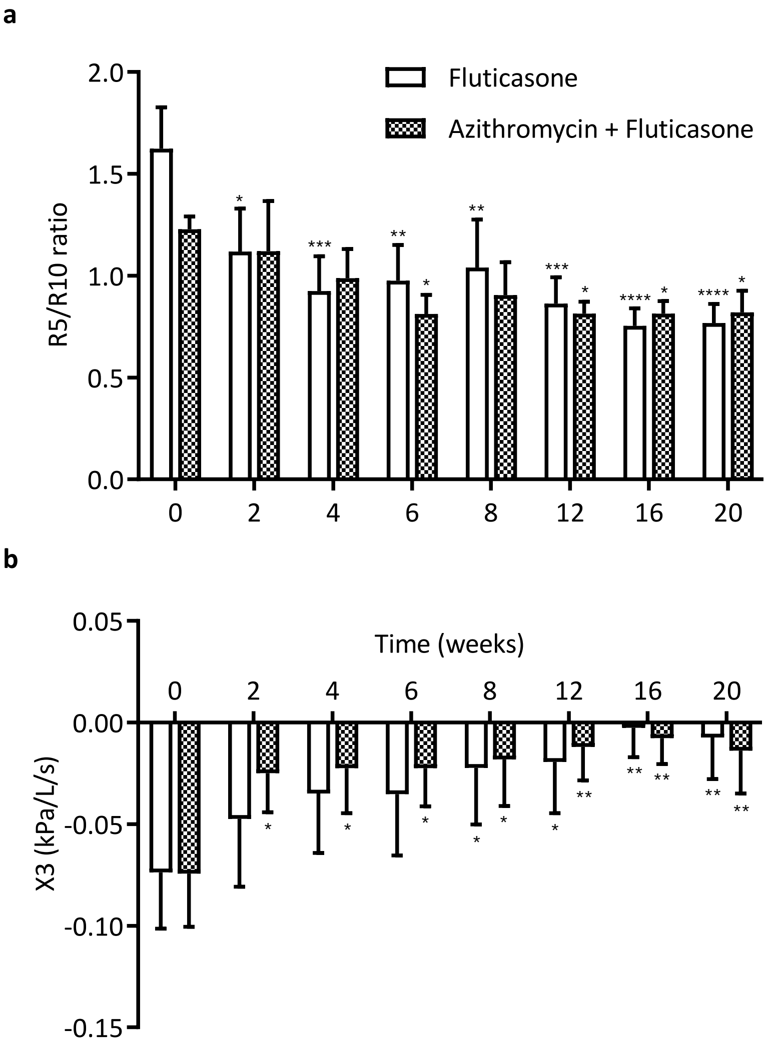

Supplementary figure 2. Gene expression in bronchoalveolar lavage fluid cells. mRNA expression of interleukin-1β (a) and CXCL-8 (b) (mean and standard error of the mean). A mixed-effects multivariate analysis was used because of a missing datum from one horse at W8. There was a significant time main effect but no group difference for interleukin-1β values ($p = 0.003$). * $p < 0.05$, ** $p < 0.01$ compared to baseline values with Dunnett's multiple comparison tests. GAPDH, glyceraldehyde 3-phosphate dehydrogenase.

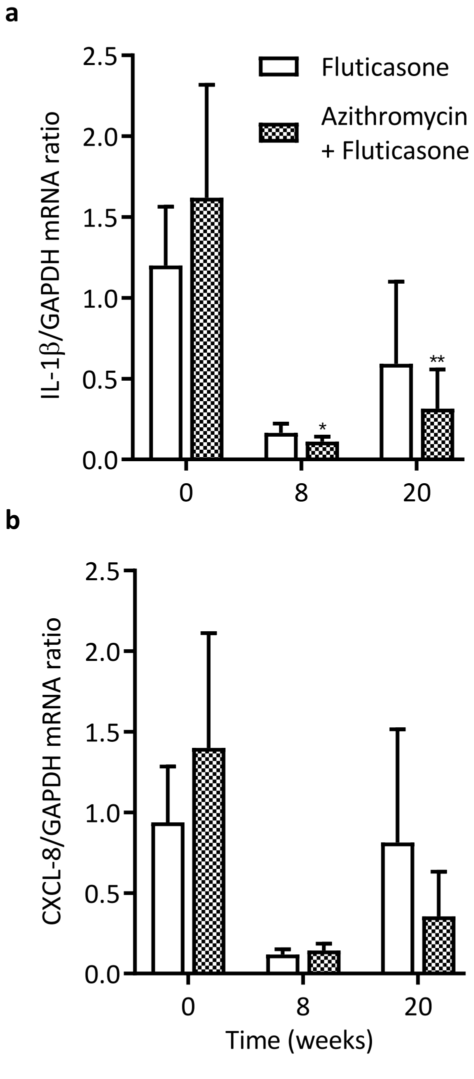

Supplementary figure 3. Gene expression in peripheral polymorphonuclear cells. mRNA expression of interleukin-1β (a) and CXCL-8 (b) (mean and standard error of the mean). There was a significant time main effect but no group difference with the two-way ANOVA for CXCL-8 values ($p = 0.006$) * $p < 0.05$, ** $p < 0.01$ compared to baseline values with Dunnett's multiple comparison tests. GAPDH, glyceraldehyde 3-phosphate dehydrogenase.

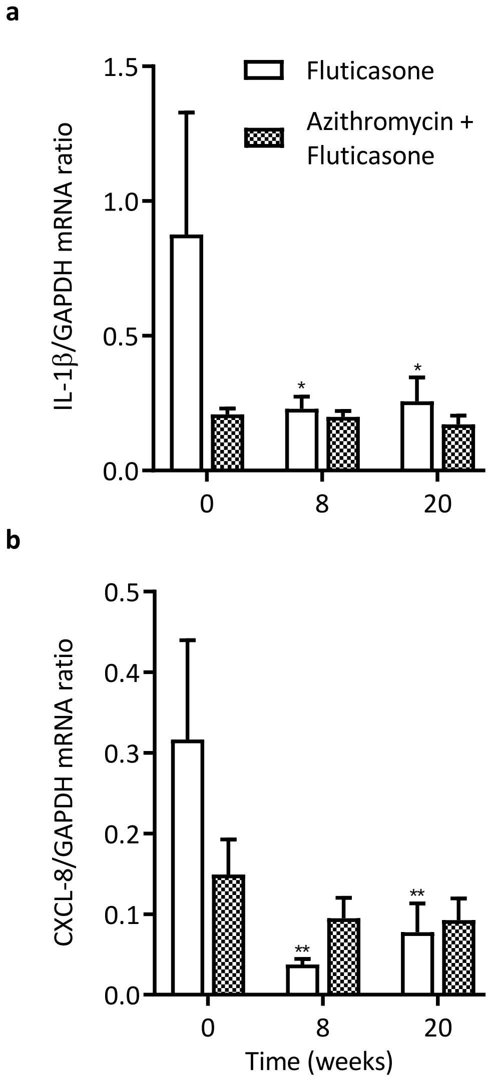

Supplementary figure 4. Gene expression in endobronchial biopsies. mRNA expression of interleukin-1β (a), CXCL-8 (b) and of the SMMHC (smooth muscle myosin heavy chain) (+) insert isoform to total myosin ratio (c) (mean and standard error of the mean). There was a significant time main effect but not group difference with the two-way ANOVA for interleukin-1β ($p = 0.001$), CXCL-8 ($p = 0.01$) and SMMHC ratio ($p = 0.002$). * $p < 0.05$, ** $p < 0.01$ compared to baseline values with Dunnett's multiple comparison tests. GAPDH, glyceraldehyde 3-phosphate dehydrogenase.

a

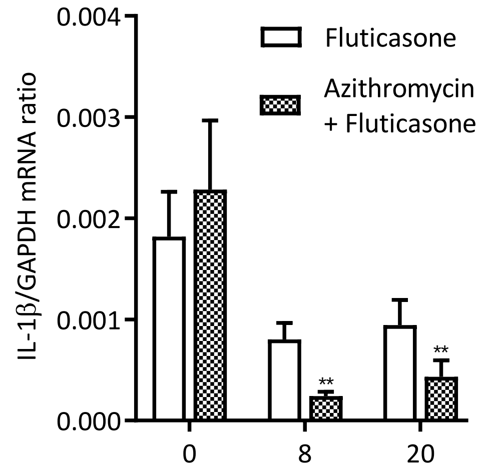

IL-1β/GAPDH mRNA ratio
0.004
0.003
0.002
0.001
0.000
Fluticasone
Azithromycin
+ Fluticasone
**
**
0
8
20

b

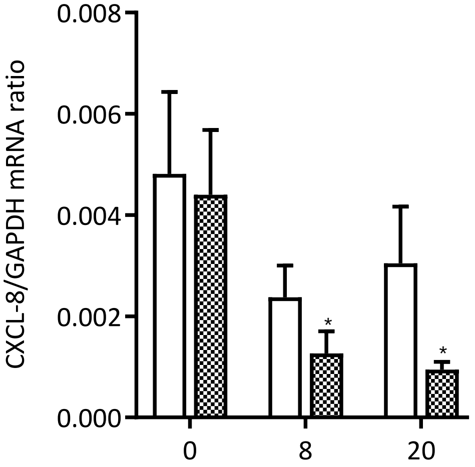

CXCL-8/GAPDH mRNA ratio
0.008
0.006
0.004
0.002
0.000
*
*
0
8
20

c

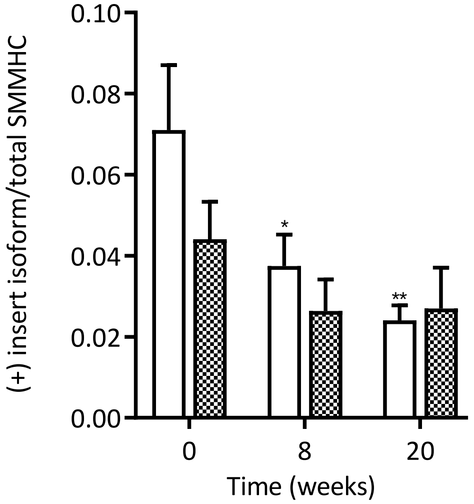

(+) insert isoform/total SMMHC
0.10
0.08
0.06
0.04
0.02
0.00
*
**
0
8
20
Time (weeks)

Supplementary table

Supplementary table 1 – Percentage of bronchoalveolar lavage fluid recovered and total cell counts (mean ± standard error of the mean)

	Bronchoalveolar lavage fluid recovered (%)		Total cell counts (*10^6)	
**Time (weeks)**	**Fluticasone**	**Fluticasone + azithromycin**	**Fluticasone**	**Fluticasone + azithromycin**
**Baseline**	36.9 ± 2.4	43.9 ± 6.8	72.9 ± 19.8	200.7 ± 120.0
**2**	55.8 ± 4.9*	51.2 ± 7.4	252.8 ± 60.1	91.2 ± 18.1
**4**	44.4 ± 4.7	56.3 ± 4.0	97.5 ± 16.8	181.6 ± 44.1
**6**	57.0 ± 3.0*	68.8 ± 4.7***	345.4 ± 77.0**	155.3 ± 39.9
**8**	51.3 ± 3.7	56.5 ± 5.9	147.3 ± 16.8	154.7 ± 47.5
**12**	61.8 ± 3.8**	59.3 ± 3.8	222.8 ± 74.5	203.7 ± 47.8
**16**	58.6 ± 5.9**	50.1 ± 8.4	209.4 ± 46.8	83.7 ± 29.9
**20**	53.8 ± 10.3	56.4 ± 5.8	167.1 ± 34.1	150.1 ± 40.0

* $p < 0.05$, ** $p < 0.01$, *** $p < 0.001$ compared to baseline values with Dunnett's multiple comparison tests.
